# A selective kernel-based cycle-consistent generative adversarial network for unpaired low-dose CT denoising

**DOI:** 10.1093/pcmedi/pbac011

**Published:** 2022-05-25

**Authors:** Chaoqun Tan, Mingming Yang, Zhisheng You, Hu Chen, Yi Zhang

**Affiliations:** National Key Laboratory of Fundamental Science on Synthetic Vision, Sichuan University, Chengdu 610065, China; College of Computer Science, Sichuan University, Chengdu 610065, China; National Key Laboratory of Fundamental Science on Synthetic Vision, Sichuan University, Chengdu 610065, China; National Key Laboratory of Fundamental Science on Synthetic Vision, Sichuan University, Chengdu 610065, China; College of Computer Science, Sichuan University, Chengdu 610065, China; College of Computer Science, Sichuan University, Chengdu 610065, China

**Keywords:** cycle-consistent adversarial network, selective kernel networks, unsupervised low dose CT, image denoising, clinical dataset

## Abstract

Low-dose computed tomography (LDCT) denoising is an indispensable procedure in the medical imaging field, which not only improves image quality, but can mitigate the potential hazard to patients caused by routine doses. Despite the improvement in performance of the cycle-consistent generative adversarial network (CycleGAN) due to the well-paired CT images shortage, there is still a need to further reduce image noise while retaining detailed features. Inspired by the residual encoder–decoder convolutional neural network (RED-CNN) and U-Net, we propose a novel unsupervised model using CycleGAN for LDCT imaging, which injects a two-sided network into selective kernel networks (SK-NET) to adaptively select features, and uses the patchGAN discriminator to generate CT images with more detail maintenance, aided by added perceptual loss. Based on patch-based training, the experimental results demonstrated that the proposed SKFCycleGAN outperforms competing methods in both a clinical dataset and the Mayo dataset. The main advantages of our method lie in noise suppression and edge preservation.

## Introduction

X-Ray computed tomography (CT) as a common non-invasive radiological diagnostic method is widely known to have potential for the examination of various diseases such as pneumonia, tumor, infarction and bleeding.^1,2^ The important role of CT in following-up on the effects on lung tissue has been widely recognized for clinical diagnosis and monitoring of COVID-19.^3^ One increasing concern about CT is the threat of excessive radiation dose. Research reducing CT dose under the guiding principle of ALARA (as low as reasonably achievable) has aroused strong attention.^4^ The universal and effective strategy to minimize the risk is to obtain low dose CT (LDCT) by decreasing the tube current of the X-ray tube and shorten the exposure time during shooting.^5^ However, lowering radiation dose inevitably increases artifacts and causes noise in reconstructed images, which could degrade the signal-to-noise ratio and affect the judgment performance. Thus, how to improve the image quality for LDCT has become a significant topic in the field of image denoising.

To date, algorithms generally include sinogram domain filtration,^6,7^ iterative reconstruction (IR),^8,9^ and image processing.^10,11^ The common sinogram filtering methods are difficult to perform on transparent raw data from commercial scanners before image reconstruction, which can lead to resolution loss and edge blurring. Simultaneously, these methods may induce artifacts in the generated image during data processing. In comparison, IR has contributed greatly to the field of LDCT. These algorithms optimize an objective function that incorporates an accurate system model, a statistical noise model, and prior information in the image domain. The common algorithms include total variation and its variants,^12–14^ dictionary learning,^15,16^ low-rank^17^ and so on. These iterative reconstruction algorithms greatly improve image quality but images may still lose some detail and suffer from remaining artifacts. Also, they require a high computational cost, which is a bottleneck in practical applications.

As an effective alternative, image post-processing has advantages with regard to non-essential raw data and efficiency. Due to developments in artificial intelligence and deep learning (DL),^18–21^ DL-based algorithms have attracted extensive attention to learn the mapping pixels-to-pixels for the corresponding routine-dose image by training with pairs of low-dose images and matched high-dose CT data. Kang *etal*.^22^ have proposed the DL-model combined with wavelet transform to effectively suppress noise and artifacts in LDCT, but a long training time is required. Chen *et al*.^23^ used the classical residual encoder–decoder convolutional neural network (RED-CNN) for LDCT denoising, and five-layer to simplify network structure and to outperform the state-of-the-art methods. Noise reduction in the above-mentioned end-to-end network notwithstanding, mean square error (MSE)-based methods have usually oversmoothed the subtle structural details by minimized per-pixel MSE. Therefore, the generative adversarial network (GAN) is used to overcome these limitations.^24^ Wolterink *et al*.^25^ have applied GAN to achieve noise supersession in LDCT. Yang *et al*.^26^ have introduced the Waserstin distance to design WGAN-GP to better retain feature information in LDCT, simultaneously, perceptual loss is used to optimize the loss function. Du *et al*.^27^ have brought a visual attention mechanism into GAN to propose VAGAN, which focuses on the information needed to achieve satisfactory performance and effectively suppress noise. You *et al*.^28^ have proposed a semi-supervised network, which achieves the generation of high-resolution CT from low-resolution CT. In general, GAN has obtained widespread interest by generating different models in LDCT.

Despite the remarkable improvement in performance, well-paired CT images for supervised training are difficult to obtain in clinical practice. Furthermore, due to the potential mode-collapsing characteristics of GAN, redundant features could be generated to affect the accuracy of diagnosis in clinical practice. Thus, unsupervised learning has caused public concern regarding unmatched-pair LDCT images. The cycle-consistent generative adversarial network (CycleGAN) is an image-to-image conversion algorithm, which is composed of two generators and two discriminators to achieve cycle-consistency by performing input-to-target image domain translation without well-paired data.^29^ However, unsupervised LDCT denoising using CycleGAN is not very effective in noise suppression.^30–32^ In this study, we designed a novel selective feature network using CycleGAN, where the unsupervised learning model can reduce LDCT image noise by adaptively selecting features.

## Methods

### Overview of LDCT denoising model

The purpose of noise reduction is to make LDCT images as similar as possible to normal dose CT (NDCT) images. This process can be simplified to the following:

$$
\begin{eqnarray*}
{\rm G} = x \rightarrow y
\end{eqnarray*}
$$

**Figure 1.**
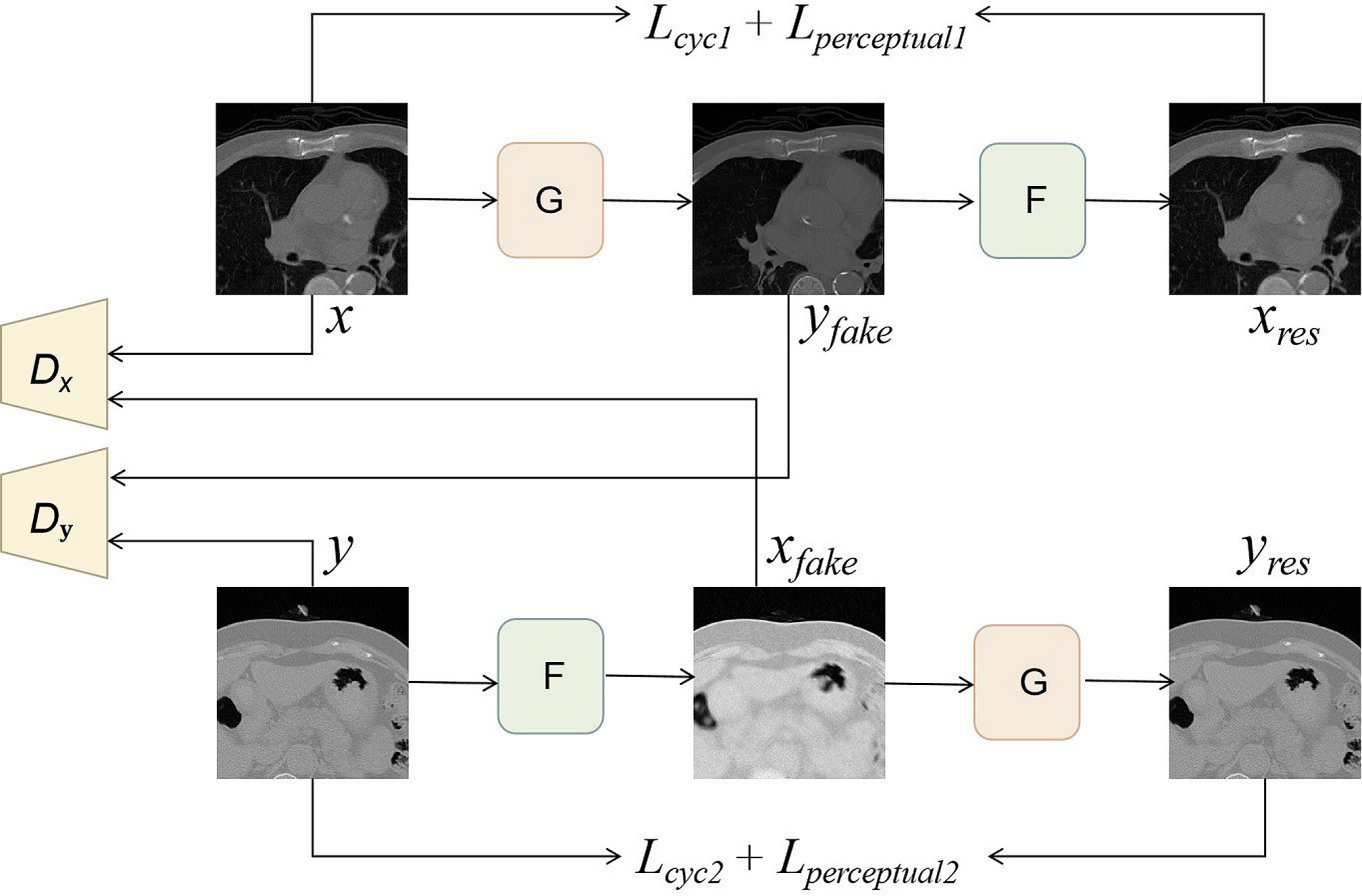
The overall framework of the CT image denoising method based on adaptive feature selection. NDCT (normal-dose CT) and LDCT images are generated by generators G and F, respectively. Discriminators *D_x_* and *D_y_* are used to distinguish which data-domain the input belongs to. Cyclic-consistency loss and perceptual loss are used to constrain the generated CT image corresponding to the input image.

Where *X* images domain is defined as LDCT data and *Y* images domain is defined as NDCT data. The overview structure of the network is shown in Fig. 1.

When LDCT *x* ($x \in X$) is input, the corresponding NDCT *y*_fake_ (${y_{fake}} \in Y$) is generated by G. Then, the corresponding LDCT *x*_res_ (${x_{res}} \in X$) is regenerated from *y*_fake_(${y_{fake}} \in Y$) by*F*. *D_x_* is used to discriminate *x* and *x*_fake_, and *D_y_* is used to distinguish *y* and *y*_fake_. The cyclic-consistency loss function^33^ is introduced to constrain the generated CT image corresponding to the input *x*. The perceptual loss^33^ function is introduced to calculate the pixel-to-pixel distance between *x* and *x*_res_. When NDCT *y* (*y*∈ *Y*) is input, the corresponding LDCT *x*_fake_(*x*_fake_ ∈ *X*) and NDCT *y*_res_ (*y*_res_ ∈ *Y*) are generated, respectively. Similarly, the cyclic-consistency loss function and the perceptual loss function are used to compute the distance between *y* and *y*_res_.

### Adaptive feature selection generator

**Figure 2.**
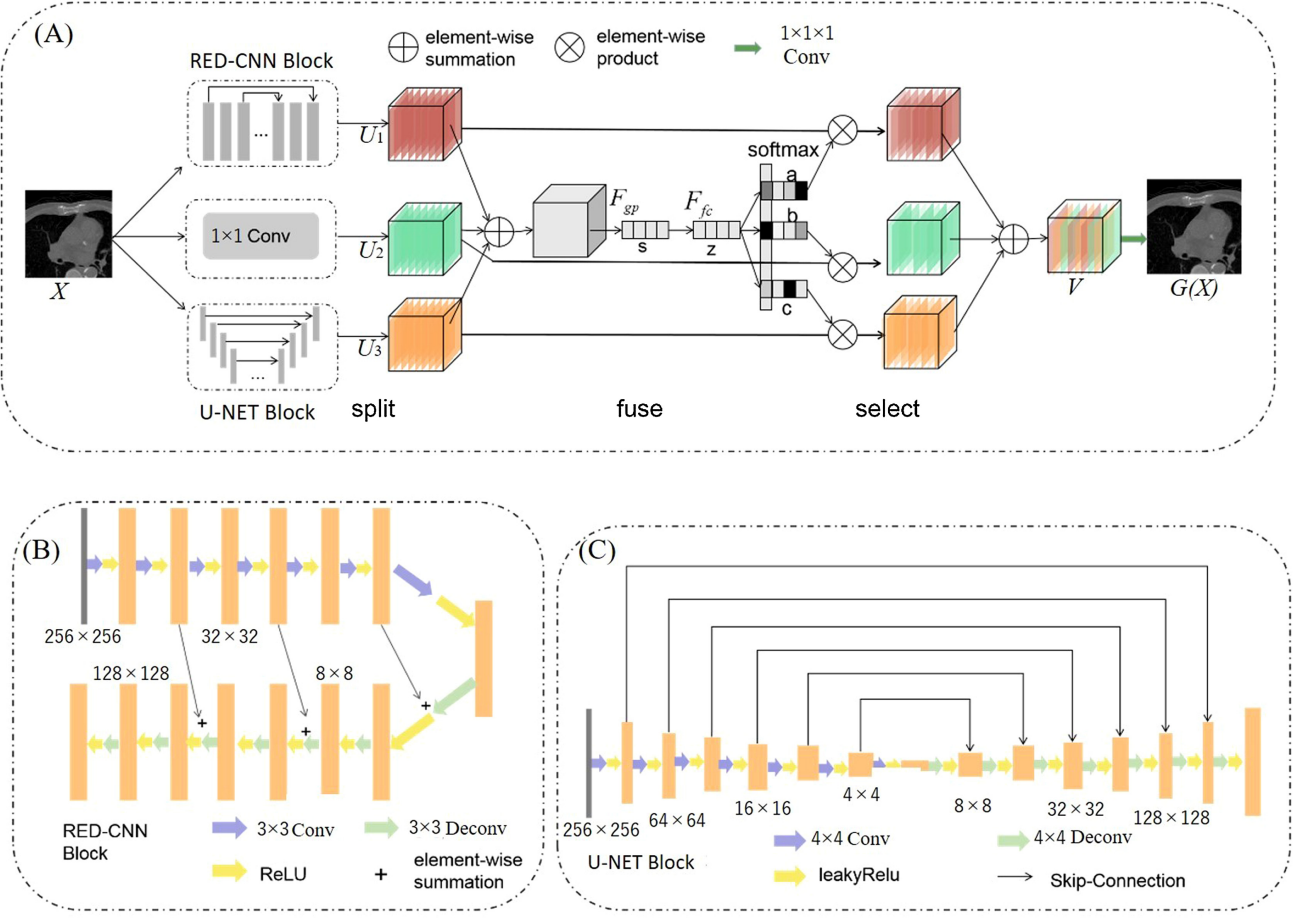
Network structure of the generator: (**A**) framework of generator, (**B**) RED-CNN block, and (**C**) U-NET block.

RED-CNN applies convolution and deconvolution instead of pooling and up-sampling, and shortcut connections which can reverse the loss of LDCT image edges and structural information. However, RED-CNN is used as the generator for unsupervised training, so noise and artifacts cannot be effectively suppressed in a clinical CT dataset. In addition, pooling and up-sampling are used to effectively remove noise and artifacts in U-NET,^34^ but result in serious loss of structural detail and cause blurred edges of images. Inspired by this, the combination of RED-CNN, U-NET, and a 1 × 1 convolutional layer as extractor, which is injected into SK-NET,^35^ is used to adaptively select features obtained from different networks with different convolution kernels. The overall structure of the proposed generator is shown in Fig. 2A. The improved RED-CNN and U-NET as a bilateral network with 1 × 1 convolutional block is used to extract the features of LDCT. The three different feature maps of ${U_1}$, ${U_2}$ and ${U_{\rm{3}}}$ are obtained by using the different convolution kernels of SK-NET. Then different feature maps are made by element-wise summation $U$ = ${U_1}$+${U_2}$+${U_{\rm{3}}}$, and encode global information through global-average pooling to generate channel-level information $S \in {R^c}$. The formation is defined as:

(1) $$
\begin{eqnarray*}
{S_c} = {F_{gp}}\left( {{U_c}} \right) = \frac{1}{{H \times W}}\sum\limits_{i = 1}^H {\sum\limits_{j = 1}^W {{U_c}\left( {i,j} \right)} } 
\end{eqnarray*}
$$

The dimensionality of *S* is reduced to produce a compact global feature $Z \in {R^{d \times 1}}$ from a fully connected layer.

(2) $$
\begin{eqnarray*}
Z = {F_{fc}}\left( S \right) = \delta \left( {B\left( {{W_S}} \right)} \right)
\end{eqnarray*}
$$

The softmax layer is used to generate three different weights *a*, *b* and *c*, where $a{}_c + b{}_c + c{}_c = 1$.

(3) $$
\begin{eqnarray*}
{a_c} = \frac{{{e^{{A_{{c^Z}}}}}}}{{{e^{{A_{{c^Z}}}}} + {e^{{B_{{c^Z}}}}} + {e^{{C_{{c^Z}}}}}}},{b_c} = \frac{{{e^{{B_{{c^Z}}}}}}}{{{e^{{A_{{c^Z}}}}} + {e^{{B_{{c^Z}}}}} + {e^{{C_{{c^Z}}}}}}},{c_c} = \frac{{{e^{{C_{{c^Z}}}}}}}{{{e^{{A_{{c^Z}}}}} + {e^{{B_{{c^Z}}}}} + {e^{{C_{{c^Z}}}}}}}\nonumber\\
\end{eqnarray*}
$$

Finally, the 1 × 1 convolutional layer is applied to obtain the output feature $V$ of CT images. The formation is defined as:

(4) $$
\begin{eqnarray*}
{V_c} = {a_c}\cdot{U_1} + {b_c}\cdot{U_2} + {c_c}\cdot{U_3}
\end{eqnarray*}
$$

Both good denoising performance and clear structural details can be gained. The details of the generator network are described as follows.

#### Abundant structural detail

To reduce the distortion of structural detail caused by up-sampling, a 1 × 1 convolution layer with 64 channels is added to better achieve cross-channel interaction and retain the integration of LDCT information. In this study, RED-CNN, U-NET, and a 1 × 1 convolutional block are used to extract the features of LDCT, and are split into three different feature maps of the same size 256 × 256 × 64.

#### Adaptive feature selection

Due to the redundancy of features extracted from different networks, the effective features can be selected in this study. Inspired by SK-NET, based on split, fuse, and select, a network that can adaptively select the features obtained by different neural networks is designed. Not only can the size of the receptive field of different convolution kernels be adjusted, but the features extracted by different networks can be effectively fused.

#### Improved RED-CNN and U-NET

The network structure of RED-CNN is shown in Fig. 2B. It consists of 14 layers, including 7 convolutional and 7 deconvolutional symmetrical layers. The convolutional and deconvolutional layers have the same kernel size of 3×3 and 64 channels. Rectified linear units (ReLU)^36^ are added for each layer. The shortcuts connection is matched between the convolutional and deconvolutional layers, which improves the convergence speed. U-NET is composed of 7 convolutional and 7 deconvolutional layers with the same kernel size of 4×4 and 64 channels. An ReLU is added for each layer. Feature maps of 256 × 256 × 64 are obtained. The network structure of U-NET is shown in Fig. 2C.

### Discriminator design

The discriminator scores the generated images and guides the training of the generator. Inspired by PatchGAN,^22^ the input images are mapped to a patch of size 4×4, which is the feature map obtained by the convolutional layer. ${x_{ij}}$ is the probability of belonging to NDCT, which corresponds to each patch *X* of the input image. The average of ${x_{ij}}$ is the output of the discriminator. For training of the generated NDCT, the receptive field of the discriminator can be effectively improved to retain high-definition details of images. The network structure of the discriminator is shown in Supplementary Fig. 1, see online supplementary material.

### Loss function

The overall loss function is as follows:

(5) $$
\begin{eqnarray*}
Loss = {L_{GAN}}(G,{D_Y},X,Y) + {L_{GAN}}(F,{D_X},Y,X) + \lambda {L_{cyc}}(G,F) + {L_{perceptual}}\nonumber\\
\end{eqnarray*}
$$

where ${L_{GAN}}$ is the adversarial loss, ${L_{cyc}}$ is the cycle-consistency loss, ${L_{perceptual}}$ is the perceptual loss function.

#### Adversarial loss

Adversarial loss^33^ is used to calculate the two mapping functions. The loss function is shown as follows.

(6) $$
\begin{eqnarray*}
{L_{GAN}}(G,{D_Y},X,Y) &=& {E_{y\sim Pdata(y)}}\left[ {\log {D_Y}(y)} \right]\nonumber\\
&&\quad + {E_{x\sim Pdata(x)}}\left[ {\log (1 - {D_Y}(G(x))} \right]
\end{eqnarray*}
$$

where *X* is LDCT; *Y* is NDCT; *G* is the generator for X→G(X). *D_Y_* aims to distinguish *G*(*x*) and *Y*. *G* aims to minimize the gap and maximize the difference of adversary, i.e. $\mathop {\min }\limits_G \mathop {\max }\limits_{{D_Y}} {L_{GAN}}(G,{D_Y},X,Y)$. Similarly, the function *F* is introduced to map *Y*→*F*(*X*), i.e. $\mathop {\min }\limits_F \mathop {\max }\limits_{{D_X}} {L_{GAN}}(F,{D_X},Y,X)$.

#### Cycle-consistency loss

Theoretically, adversarial loss can compute which target domain the generated output belongs to. However, with large enough capacity, the mapping of inputs to outputs in the target domain can be randomly arranged, where the mapping cannot be guaranteed to pair the output with the corresponding input. Therefore, to further constrain the matching of the generated image and decrease the data domain space of mapping functions, the cycle consistency can be loaded in the mapping functions *G* and *F*. The cycle-consistency loss^33^ is defined as

(7) $$
\begin{eqnarray*}
{L_{CYC}}(G,F) = {E_{x\sim Pdata(x)}}[{\left\| {F(G(x)) - x} \right\|_1}] + {E_{x\sim Pdata(y)}}[{\left\| {G(F(y)) - y} \right\|_1}]\nonumber\\
\end{eqnarray*}
$$

For the translation cycle of *X*→*G*(*X*)→*F*(*G*(*X*)), the cycle-consistency function can give the generated images corresponding to *X* by calculating the L1-norm between *X* and *F*(*G*(*X*)). This is called forward cycle-consistency. Similarly, the mapping functions *G* and *F* should also conform to backward cycle-consistency for the translation of *Y*→*F*(*Y*)→*G*(*F*(*Y*)).

#### Perceptual loss

Cycle-consistency loss can calculate the pixel-level distance to ensure the matching of generated image and input image; however, structural texture and details are lost. That is why the perceptual loss is added to guide the generator to learn more feature details of images. The perceptual loss^37^ is usually calculated from the feature maps of VGG-16. The perceptual loss function is defined as follows.

(8) $$
\begin{eqnarray*}
LOS{S_{perceptual}} &=& {E_{x\sim Pdata(x)}}{\left\| {\phi (x) - \phi (F(G(x)))} \right\|_1}\nonumber\\
&&\quad + {E_{x\sim Pdata(x)}}{\left\| {\phi (y) - \phi (G(F(y)))} \right\|_1}
\end{eqnarray*}
$$

where $\phi $ is the feature maps. In this section, both the second max-pooling layer with 128 channels and the last max-pooling layer with 512 channels in VGG-16 are used to calculate the perceptual loss.

### Evaluation metrics

#### Root mean square error

Root mean square error (RMSE) is used to measure the deviation between the generated CT image and NDCT. It is computed by using the arithmetic square root of MSE. The definition is shown in Eq. (9).

(9) $$
\begin{eqnarray*}
RMSE = \sqrt {MSE} ,
\end{eqnarray*}
$$

$$
\begin{eqnarray*}
MSE = \frac{1}{{mn}}\sum\limits_{i = 0}^{m - 1} {\sum\limits_{j = 0}^{n - 1} {{{[I(i,j) - K(i,j)]}^2}} } 
\end{eqnarray*}
$$

where $m \times n$ is the size of the clear image *I* and the noise image *K*.

#### Peak signal-to-noise ratio

Peak signal-to-noise ratio (PSNR) reflects the ratio between the maximum signal of the image and the noise of the image. It is an evaluation index for calculating errors between corresponding pixels. The higher the PSNR, the better the image quality. PSNR is defined in Eq. (10).

(10) $$
\begin{eqnarray*}
PSNR = 10.{\log _{10}}\left( {\frac{{MAX_I^2}}{{MSE}}} \right)
\end{eqnarray*}
$$

where $MAX_I^2$ is the maximum of pixels.

#### Structural SIMilarity

Structural SIMilarity (SSIM) is applied to judge the similarity between *X* and *Y*, based on the brightness, contrast, and structure of images. SSIM is defined in Eq. (11).

(11) $$
\begin{eqnarray*}
SSIM(x,y) = \frac{{(2{\mu _x}{\mu _y} + {c_1})(2{\sigma _{xy}} + {c_2})}}{{(\mu _x^2 + \mu _y^2 + {c_1})(\sigma _x^2 + \sigma _y^2 + {c_2})}}
\end{eqnarray*}
$$

where ${\mu _x}$, ${\mu _y}$ are the mean of *x*, *y*; $\sigma _x^2$, $\sigma _y^2$ are the variances of *x* and *y*, respectively; ${\sigma _{xy}}$ is the covariance of *x*, *y*; ${c_1} = {( {{k_1}L} )^2}$, ${c_2} = {( {{k_2}L} )^2}$ are constants, avoid division by zero, where *L* is the range of pixel values. Usually, ${k_1} = 0.01$, ${k_{\rm{2}}} = 0.0{\rm{2}}$ and ${c_3} = {c_2}/2$.

#### Qualitative evaluation

In addition to quantitative indicators, a qualitative indicator may also be needed to evaluate the quality of the generated CT image. Since CT images are to help clinicians make pathological diagnoses, the denoising results of LDCT are judged by the subjective senses of two professionals.

## Experimental design and results

### Experimental datasets

#### Clinical dataset

A real unmatched-pair clinical database was applied for evaluating the performance of the proposed model. There were two different 512 × 512 LDCT images, 10 ma and 30 ma respectively. 160ma is the dose of NDCT image in both datasets. This clinical dataset was the main problem to be solved. The specific parameters of two different LDCT images are shown in Supplementary Table 1, see online supplementary material.

Due to the diversity of the human body, different window widths are required for observing and displaying CT images. Window widths of 0.2–0.28 and 0–0.33 were selected, respectively, where 0.2–0.28 was used for observing the tissue and 0–0.33 was used for observing the lung. Some typical CT images are shown in Supplementary Fig. 2 (see online supplementary material). The 10 ma LDCT dataset was composed of 337 pairs of LDCT and NDCT images from 6 anonymous patients. In our experiments, 218 pairs of LDCT and NDCT images from 5 patients were randomly selected for training and the remaining pairs were used for testing. To effectively train the network, patch-based extraction was performed to obtain CT images with local details required for denoising training and to increase the number of samples. All CT images were cropped into a 256 × 256 patch every 16 pixels, and 81 209 pairs of 256 × 256 LDCT and NDCT images were obtained.

The 30 ma LDCT dataset contained 562 pairs of LDCT and NDCT images from 10 anonymous patients. A total of 498 pairs of LDCT and NDCT images from 9 patients were randomly selected for training and the remaining pairs were used for testing. Similarly, 143 922 pairs of 256 × 256 LDCT and NDCT images were obtained.

#### Mayo dataset

Mayo,^38^ a publicly available dataset with paired NDCT and LDCT images, was created in “the 2016 NIH-AAPM-Mayo Clinic Low Dose CT Ground Challenge” to evaluate the performance of LDCT denoising algorithms. The dataset included 5936 NDCT images with 512 × 512 and quarter-dose simulated LDCT images from 10 patients. In this paper, 135 250 pairs of 256 × 256 CT image patches from 9 patients were randomly extracted as the training set, and the remaining data from 1 patient was used as a testing set. For the research of unsupervised LDCT image denoising, all pairs were in disorder. This was used to validate the effectiveness of the proposed model.

### Training parameter

In this study, the model was optimized by the Adam^39^ algorithm. The initial learning rate was 10^-5^ which decreased linearly from iterating 100 000 steps. It remained unchanged until it was reduced to 10^-7^ at 700 000 steps. The weight parameter $\lambda $ of the generator was set to 10 and the batch size was set to 1. The experiment was based on Tensorflow14 and Python 3.6. The model was trained on a PC (Intel i7 processor and 11G video memory) with a graphics processing unit card (Nvidia 2080TI). The final model was obtained when 1 million steps was reached.

The experiments were performed on two different LDCT datasets. Multiple different state-of-the-art algorithms were compared with ours in the clinical dataset, including BM3D,^40^ K-SVD,^41^ and unsupervised models CCADN^30^ and CycleGAN. Moreover, BM3D, K-SVD, supervised methods such as RED-CNN^23^ and Q-AE,^42^ and unsupervised algorithm CycleGAN and CCADN were compared with ours on Mayo dataset. Due to the unpaired clinical dataset, it was impossible to calculate quantitative indicators such as PSNR, RMSE, and SSIM. Therefore, qualitative evaluations were mainly carried out by the visual sense of experts.

### Experimental results

#### Clinical dataset

##### 0.2-0.28 window width

**Figure 3.**
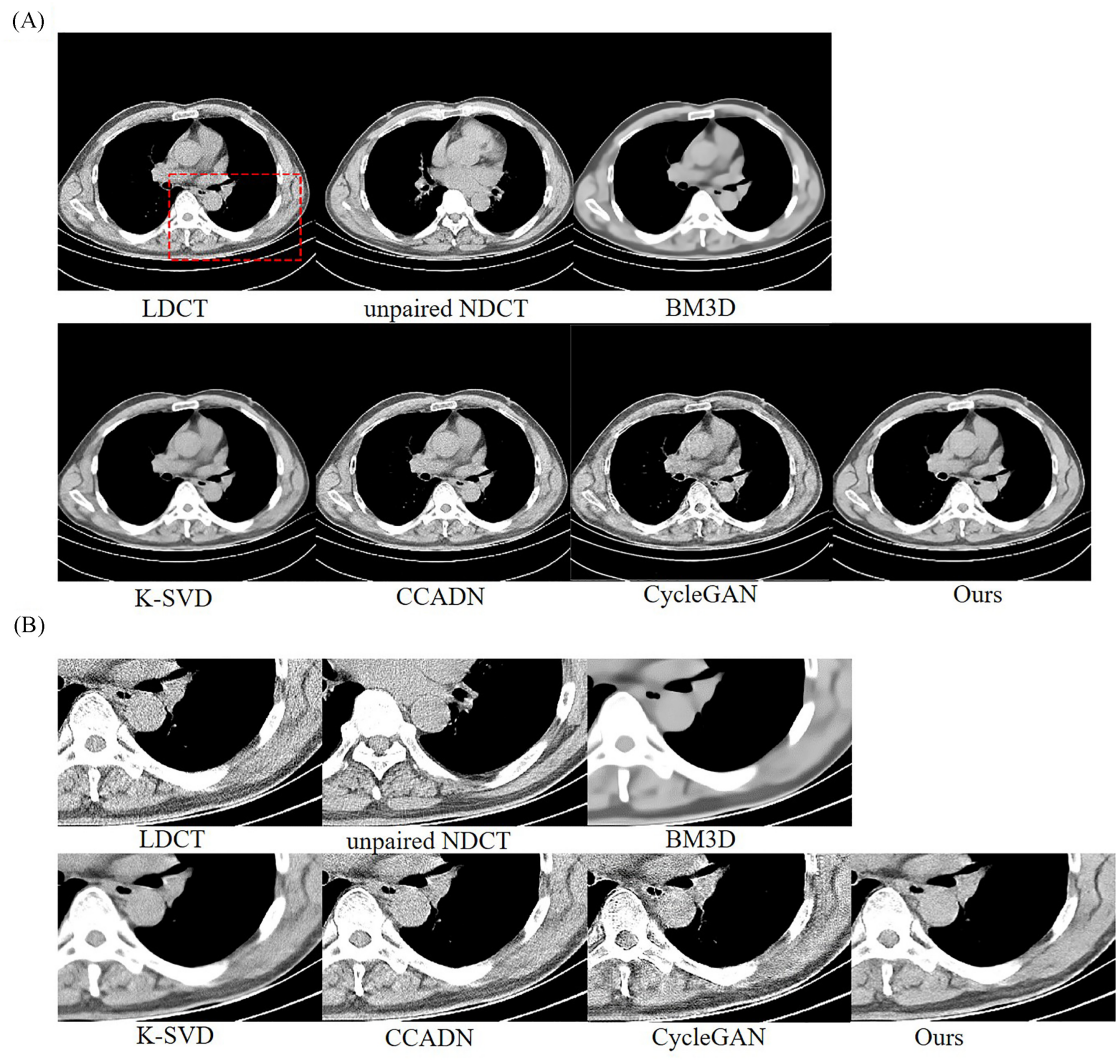
Results and magnified images over a ROI of a 30 ma dataset with 0.2–0.28 window width for comparison. The BM3D, K-SVD, and unsupervised algorithms CCADN and CycleGAN have been compared in this study. The area indicated by a red rectangle is the ROI that was magnified to show the experimental results.

Two LDCT images were used to test the performance of the model, with window widths of 0.2–0.28 and 0–0.33, respectively. The experimental results and the magnified images for a region of interest (ROI) with window width of 0.2–0.28 are shown in Fig. 3A and B. The tissue can be observed, i.e. the areas with higher gray values. For the LDCT of Fig. 3A, image noise and artifacts exist near the structure with a high attenuation coefficient. All methods suppressed image noise to different extents. For multiple comparison results, our method has achieved the best experimental results.

However, other methods could not effectively suppress noise or cause blurring of CT images to various degrees. As seen in Fig. 3A, the noise of LDCT could be effectively suppressed for BM3D, but the generated CT images were so blurred that there was serious loss of structural details. K-SVD can also cause blurring of edges and a good quality CT image is not obtained. Compared with traditional algorithms, the unsupervised algorithm CCADN achieved better denoising, but there was still noise and artifacts. It can be seen, particularly from Fig. 3B, that with CCADN and GycleGAN the noise was not effectively suppressed and artifacts in the generated CT images could cover the local details of the images. However, it is clear that compared to the above-mentioned algorithms, the noise was more effectively suppressed and clearer structural features are retained.

##### 0-0.33 window width

**Figure 4.**
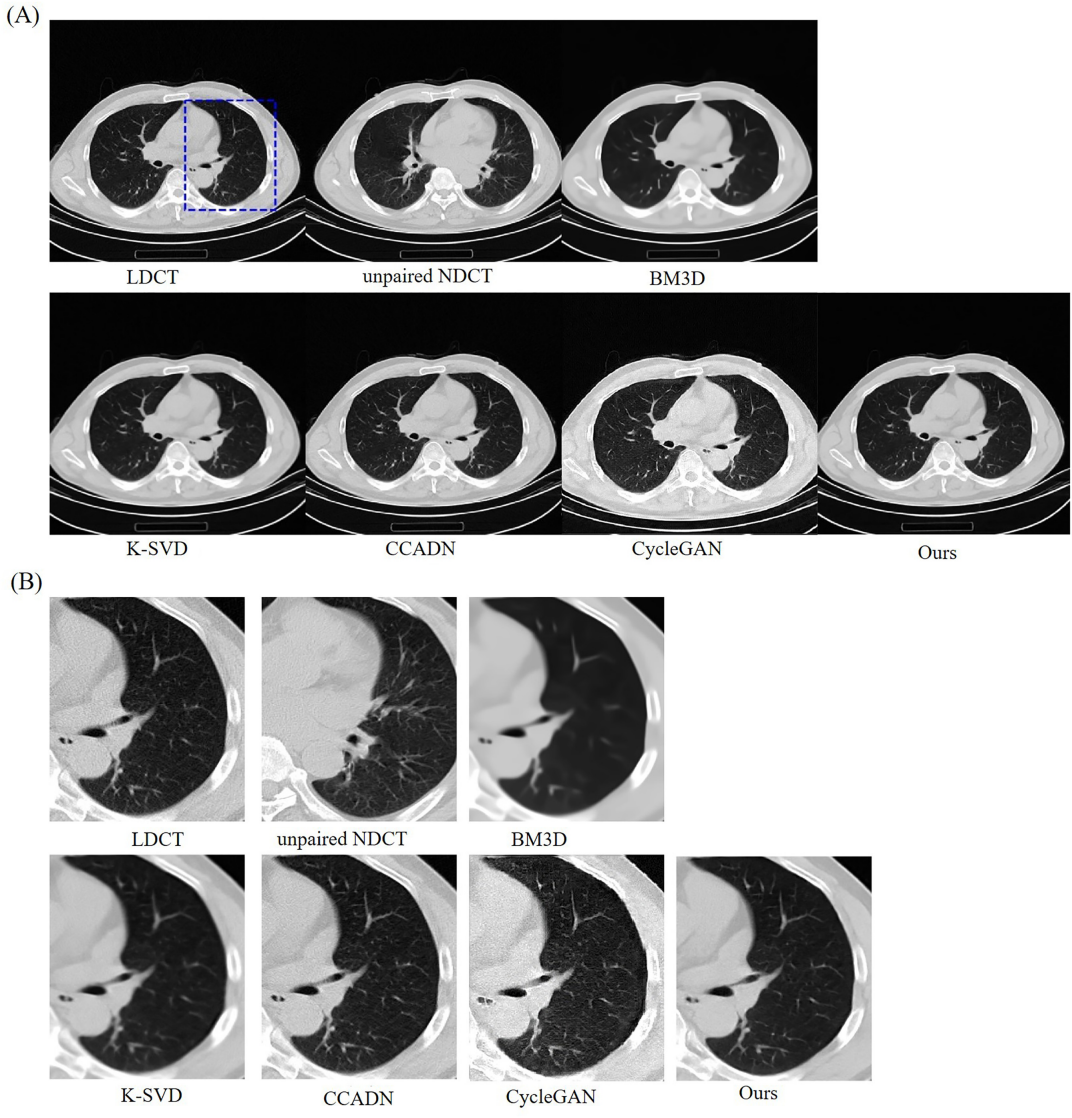
Results and magnified images of a ROI of a 30 ma dataset with 0–0.33 window width for comparison. The BM3D, K-SVD, and unsupervised algorithms CCADN and CycleGAN were compared in this study. The black area indicated by the blue rectangle is the ROI that was magnified to show the experimental results.

CT images of lungs could be observed with a window width of 0–0.33, i.e. the black area marked by the blue rectangle in Fig. 4. The results were similar to the training results with the 0.2–0.28 window width. It can be concluded that ours achieved the best denoising performance compared with other algorithms, and the best quality of CT image was generated. The experimental results and magnified images of an ROI based on the different algorithms are shown in Fig. 4A and B.

Although most image noise and artifacts were eliminated, the structural details were smoothened by BM3D and K-SVD. Meanwhile, for the unsupervised learning algorithms CCADN and CycleGAN, obviously, there was still more noise and artifacts compared with ours, especially for the results based on CycleGAN. SKFCycleGAN can generate a clear lung CT image.

Usually, the five metrics including noise suppression, artifact reduction, lesion discrimination, contrast retention, and overall quality are used for subjective evaluation by doctors (5 is best, 1 is lowest). On the basis of the different algorithms, two radiologists with 6 years of clinical experience respectively provided their scores. The unpaired NDCT images were references, and the average scores of the two experts were used as final results. The statistical results are shown in Supplementary Table 2, see online supplementary material. From the five indicators, all of the methods could suppress LDCT noise and effectively reduce artifacts, but our algorithm produced better scores than the other methods.

#### Mayo dataset

**Figure 5.**
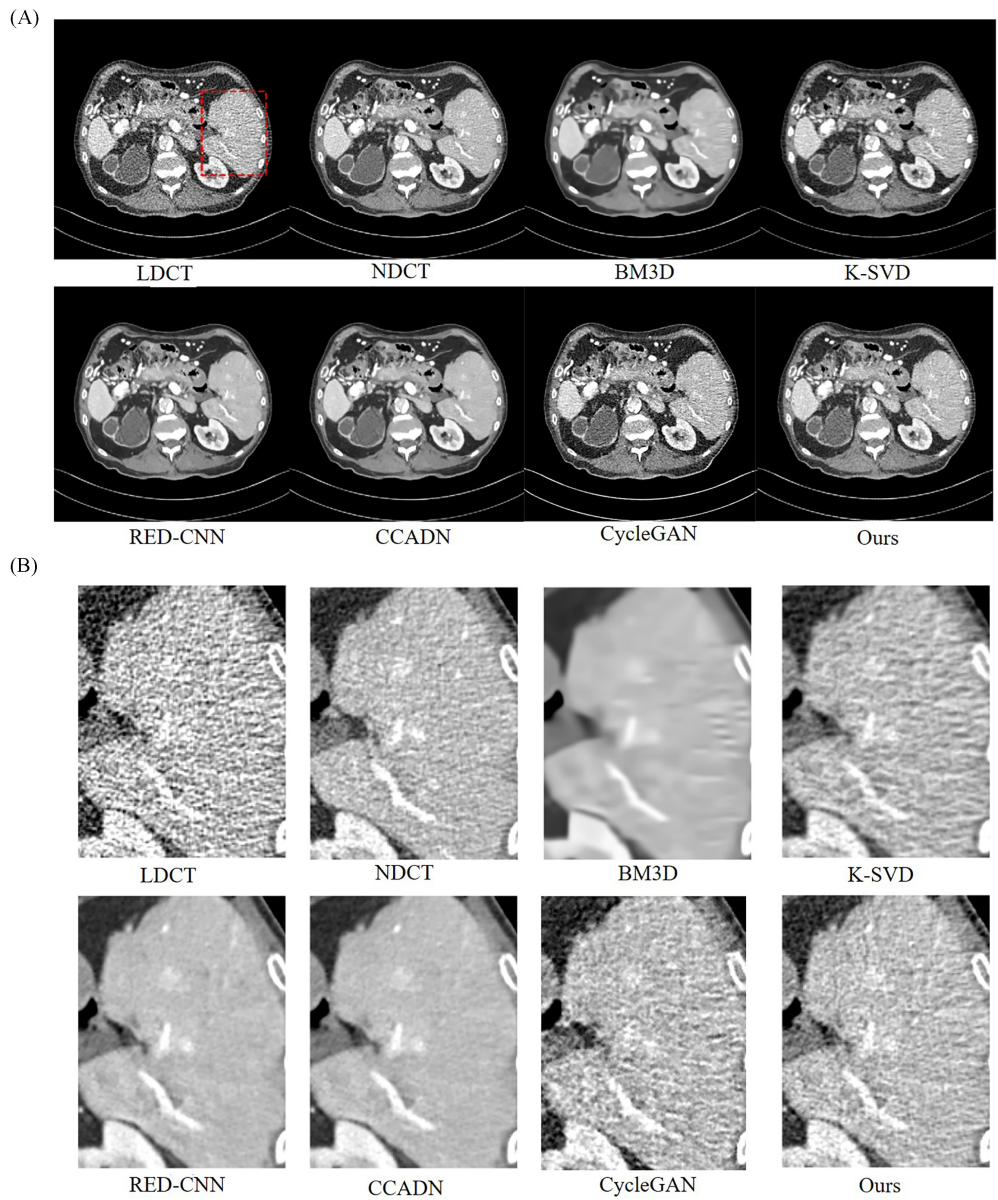
Results of the Mayo dataset and a magnified ROI for comparison of the BM3D, K-SVD, RED-CNN, CCADN and CycleGAN algorithms used in this study. The area indicated by a red rectangle is the ROI that was magnified to show the experimental results.

To better verify the robustness and generalization of the proposed method, it was tested on the Mayo dataset and compared with the traditional algorithms such as BM3D and K-SVD, the classical supervised learning algorithms RED-CNN and Q-AE, and the unsupervised learning algorithms CycleGAN and CCADN. The experimental results on the Mayo dataset are shown in Fig. 5.

For Fig. 5A, the denoising result of BM3D was over-smoothed, and noise and artifacts were not effectively suppressed in the K-SVD image. Even if RED-CNN gave the best performance, our method had better denoising results compared to traditional algorithms, CCADN and CycleGAN. The results and the magnified ROI for the Mayo dataset are shown in Fig. 5B.

In the magnified ROI of BM3D, the experimental results obtained still presented too blurred CT images. Since MSE-based algorithm is usually trained by minimized per-pixel MSE, the generated CT has higher quantitative value and is closer to NDCT images. For the unsupervised learning algorithm, there was a lack of matched LDCT and NDCT images, thus denoising results could only be obtained and compared visually. A comparison of the quantitative results obtained using the different methods is shown in Table 1.

**Table 1.** Comparison of quantitative results associated with the Mayo dataset.

Model	RMSE	PSNR	SSIM
BM3D	0.0097	40.23	0.9385
K-SVD	0.1284	37.83	0.9455
RED-CNN	0.0065	43.71	0.9686
CCADN	0.0089	41.09	0.9471
CycleGAN	18.8017	22.65	0.8469
**Ours**	**0.0085**	**41.45**	**0.9535**

It can be seen that the MSE-based supervision learning methods had the best evaluation metrics compared with other models. RMSE, PSNR, and SSIM were also calculated by MSE. Compared with traditional algorithms and unsupervised learning algorithms, the best performance was obtained for RMSE, PSNR, and SSIM with our algorithm, with values of 0.0085, 41.45 and 0.9535, respectively.

#### Different model and performance trade-offs

**Figure 6.**
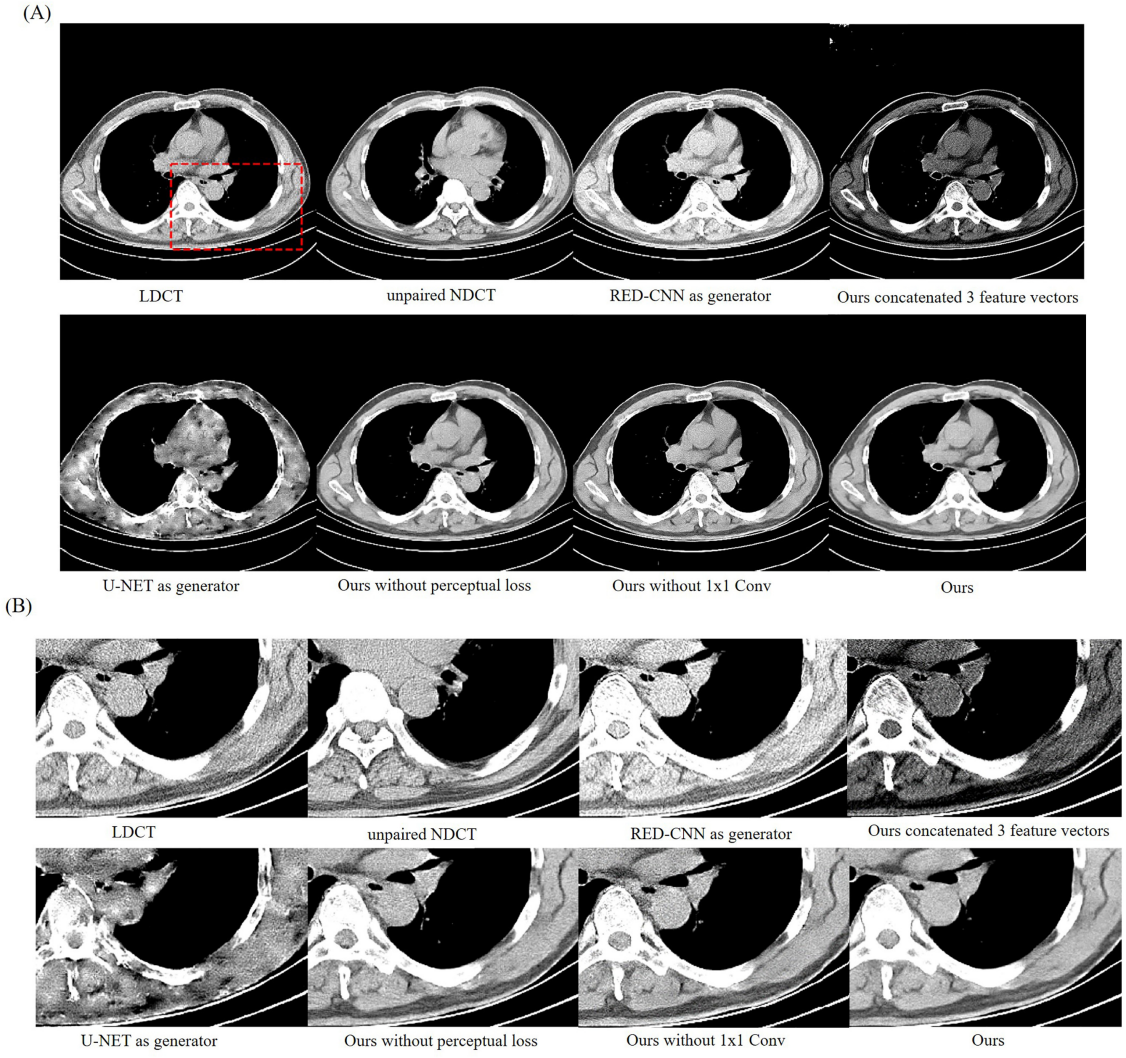
Experimental results and the magnified ROI of the different models. The four different models were compared, including a generator with only RED-CNN, a generator with only U-NET, and a generator with concatenated three-feature vectors and without a 1 × 1 convolution block. A model without perceptual loss function was also examined to prove the availability of the added perceptual loss function. To ensure fairness, the other parameters remain unchanged in the comparison. The area indicated by a red rectangle is the ROI that was magnified to show the experimental results.

In these experiments, to verify the effectiveness of designed generator networks, ours was compared with four different models, including a generator with only RED-CNN, a generator with only U-NET, and a generator with concatenated three-feature vectors and without a 1 × 1 convolutional block. In addition, a model without a perceptual loss function was also examined to prove the availability of the added perceptual loss function. To ensure fairness, the remaining parameters remain unchanged in the comparison. The experimental results and the magnified ROI for the different models are shown in Fig. 6.

In Fig. 6B, the generated CT images obtained still had noise, and artifacts were introduced. CT image contrast was reduced only for RED-CNN as the generator. Detailed features of the generated CT image were severely lost when the improved U-NET was used as generator. Some artifacts appeared when the three-feature vectors were directly concatenated. Additionally, using our designed network without perceptual loss, the noise could not completely be removed and a certain amount of blurring was produced. The 1 × 1 convolutional layer was added to achieve better cross-channel correlation and retain the integration of LDCT information. It also introduced more nonlinearity and improves generalization ability. It is clear that there was still noise and distortion of structural detail without the 1 × 1 convolutional block. Finally, our method effectively suppressed noise and artifacts, retained more structure and the edges of detailed features, and outperformed competing models.

#### Different dose and performance trade-offs

**Figure 7.**
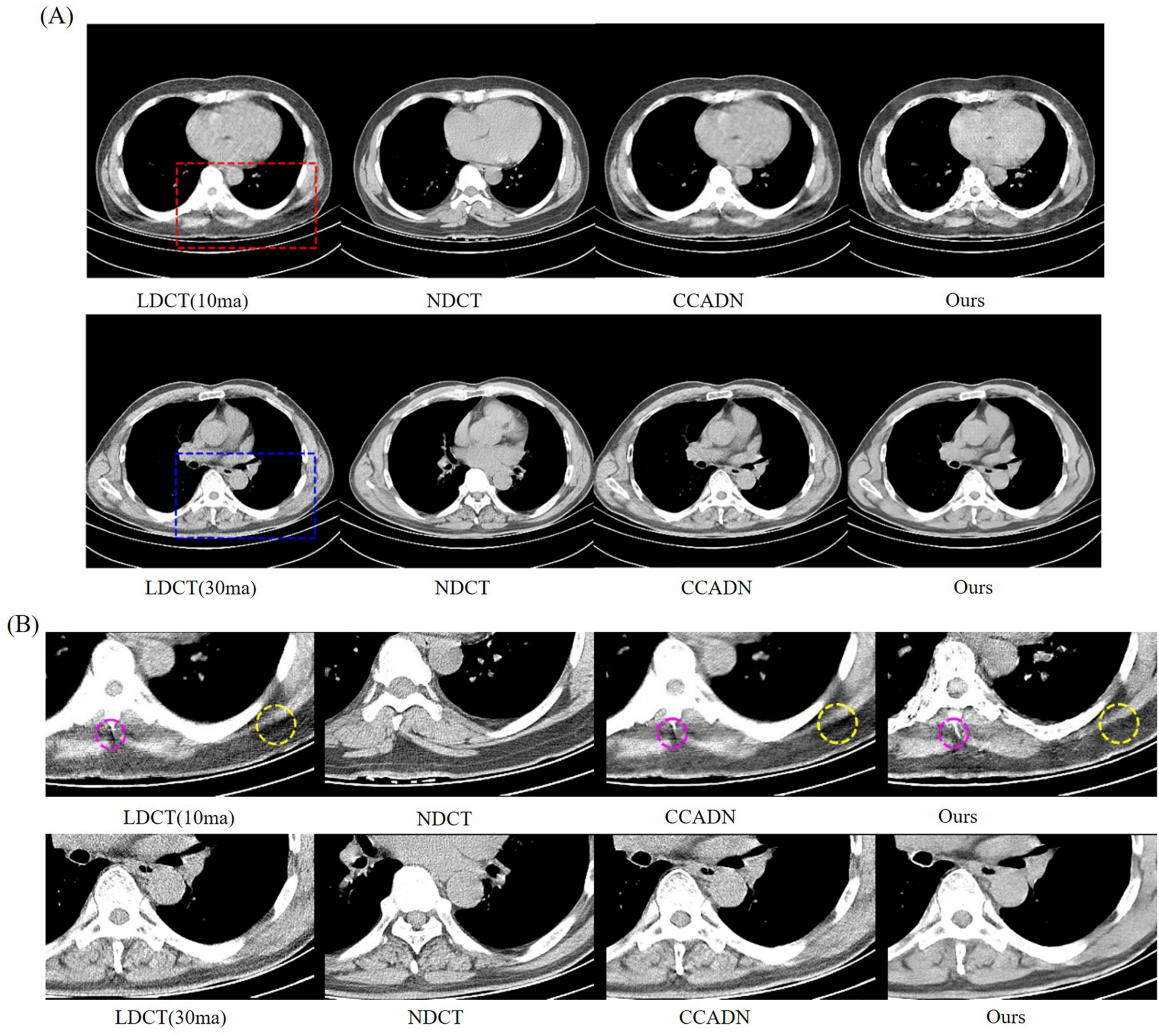
The experimental results and magnified parts of different 10 ma and 30 ma dose datasets for comparison. CCADN have been compared with ours in this paper. The red and blue rectangles indicate the magnified images of an ROI of 10 ma and 30 ma dose datasets, respectively. More structural details can be seen, as highlighted by the yellow and purple circles.

Based on the reduced dose, ensuring the completeness of the image details as much as possible is suitable for clinical practice. In clinical datasets, 10 ma and 30 ma images have differences for the preservation of detailed information and the degree of noise. In this paper, 30 ma LDCT images were used as the main research data, and 10 ma LDCT images were tested to verify the significance of the proposed method. The unsupervised learning algorithm CCADN was compared with ours. Discussion of different doses of CT images has also demonstrated the applicability of our method. The experimental results and the magnified ROI of different doses are shown in Fig. 7.

From Fig. 7B it can be seen that our method achieved good denoising results on the 10 ma CT dataset, and existing structural details were properly retained, as indicated by yellow and purple circles. Compared with 30 ma LDCT, 10 ma data was processed with noise reduction, and although lost features were restored to a certain extent, there was still detail loss. For 30 ma LDCT data, CT images with completely detailed information is obtained after denoising processing. In clinical diagnosis, excessive loss of detailed information can lead to changes of results. Hence, 30 ma CT images were selected as the main experimental dataset to evaluate the proposed model. The proposed model could effectively reduce the harm caused by CT detection without affecting the quality of diagnosis.

## Conclusion

Considering the shortage of well-paired CT images, inspired by RED-CNN and U-Net, we propose a novel unsupervised learning model based on CycleGAN for LDCT image denoising. An adaptive feature selection generator is designed, and a patchGAN discriminator is used to generate CT images maintaining more detail, that is aided by added perceptual loss. Compared with traditional methods and other unsupervised learning algorithms, the experimental results have confirmed that our proposed model is superior with both a clinical dataset and the Mayo dataset. The main advantages of our method lie in noise suppression and edge preservation.
